# Rare Double Reciprocal Translocations in a Man with Reproductive Success and Recurrent Pregnancy Loss

**DOI:** 10.2478/bjmg-2026-0007

**Published:** 2026-10-14

**Authors:** FE Aouni, F Smaili, K Zerrouki, J Ahmidi, S Meziane, K Ahmidouch, A Chyadmi, A El ammari, H Saadi, M Tajir

**Affiliations:** Medical Genetics Laboratory, Central Laboratory, Mohammed VI University Hospital, Oujda, Morocco; Faculty of Medicine and Pharmacy, Mohammed Premier University, Oujda, Morocco; Medically Assisted Procreation Unit, Mohammed VI University Hospital, Oujda, Morocco

**Keywords:** Double reciprocal translocations, Recurrent pregnancy loss, phenotypically normal child, Karyotyping analysis, Alternate segregation, Genetic counselling

## Abstract

Double reciprocal translocations represent an extremely rare cytogenetic entity. Carriers have a significantly increased risk of producing unbalanced gametes during meiosis, which can lead to adverse reproductive outcomes such as miscarriages or offspring with congenital anomalies. Despite the high reproductive risk, they can give birth either to a balanced translocation carrier with a normal phenotype (similar to the parents) or to a chromosomally normal completely healthy infant, resulting only from alternate 2:2 segregation. Here, we report an exceptional case of double-balanced reciprocal translocations involving four chromosomes (1, 3, 15 and 18) in a phenotypically normal male partner. The couple was investigated due to a history of recurrent pregnancy loss, having already had one phenotypically normal child. The aim of this report is to analyze the cytogenetic finding, predict the possible meiotic segregation outcomes, and discuss the implications in genetic counseling.

## INTRODUCTION

Pregnancy loss is defined as a spontaneous loss of a pregnancy before 24 weeks of gestation, affecting approximately 15–25% of pregnancies [1]. Pregnancy loss can be categorized as sporadic or recurrent, depending on the number of losses and whether they happen consecutively. Most sporadic miscarriages are occurring before 10th week of gestation and result in 70% from random chromosomal abnormalities, such as trisomy, monosomy, and polyploidy [2]. However, recurrent pregnancy loss (RPL) represents a distinct disorder, defined by the loss of two or more confirmed clinical pregnancies [1]. In approximately 2–5% of couples with RPL, one of partners carries a balanced structural chromosomal abnormality, most frequently, a balanced reciprocal or Robertsonian translocation [3,4].

Carriers of balanced chromosomal translocation account for 0.08–0.3% of the general population [5]. Although, they are usually phenotypically normal, they are at a high risk of miscarriage. The presence of these chromosomal rearrangements can lead to unbalanced segregation during meiosis, producing gametes with unbalanced chromosomes, such as those with deletions or duplications. This may result in a live birth with multiple congenital malformations and/or intellectual disabilities [6,7]. Nevertheless, there is a rare possibility of having a child with a normal phenotype. In these cases, the offspring may present with a normal karyotype or may be a carrier of the same balanced translocation as the parent, as documented in several familial studies [8,9].

Double reciprocal translocations represent an extremely rare cytogenetic entity. While simple reciprocal translocation is among the most common structural rearrangements, occurring in 1 in 500 live births [5]. The coexistence of two independent translocations in the same individual is an exceptional finding, with only a few cases are reported in literature[10,11].

In this study, we reported the case of a couple referred to our laboratory for cytogenetic exploration for recurrent pregnancy loss. Despite having a healthy child, the husband’s karyotype revealed the presence of double balanced reciprocal translocations.

## CASE REPORT

We received a couple for medical genetics consultation at the Mohammed VI University Hospital Center in Oujda for cytogenetic exploration of recurrent pregnancy loss.

The unrelated couple, who had been married for 12 years, presented with a history of five consecutive miscarriages, none expending two months of gestation, and were parents to a healthy 9-year-old daughter (Figure 1). Clinical examination of both partners was normal.

**Figure 1.**
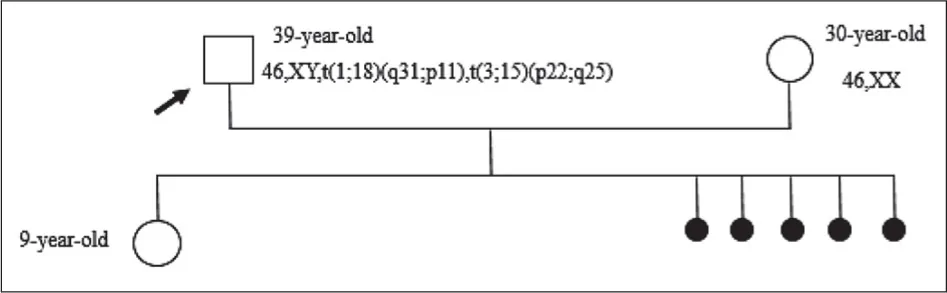
Pedigree of the couple. The proband (arrow) is a male carrier of a double reciprocal translocation. The couple has a history of recurrent pregnancy loss (indicated by spontaneous abortions).

The 39-year-old husband had no significant pathological history other than recurrent miscarriages and was healthy with normal intelligence. His 30-year-old wife also exhibited a normal phenotype.

Cytogenetic analysis was performed on peripheral blood lymphocytes isolated from the couple. Whole blood samples collected in sodium heparin were cultured for 72 hours in 10 ml of culture medium containing amino acids, vitamins, antibiotics, and fetal calf serum with phytohemagglutinin added as a mitogen. Incubation was carried out at 37°C with 5% of CO_2_. Metaphase arrest was induced by colchicine, followed by hypotonic treatment to disperse chromosomes and fixation with methanol-acetic acid. The cell suspension was spread on glass slides, the R-banding was performed by thermal denaturation and Giemsa staining. A total of 30 metaphases were analyzed at a resolution of 450/500 bands per haploid set using Ikaros® software (MetaSystems, Germany), among these, 15 metaphases were karyotyped and classified according to the latest International System for Human Cytogenomic Nomenclature 2024 (ICSN 2024)(12).

Karyotype analysis of the husband revealed a complex chromosomal rearrangement involving two independent balanced reciprocal translocations: 46,XY,t(1;18) (q31;p11),t(3;15)(p22;q25)

- A translocation between chromosomes 1 and 18, with breakpoints at q31 and p11.
- A translocation between chromosomes 3 and 15, with breakpoints at p22 and q25.

Both translocations appeared to be balanced, with no detectable loss or gain of genetic material (Figure 2).

**Figure 2.**
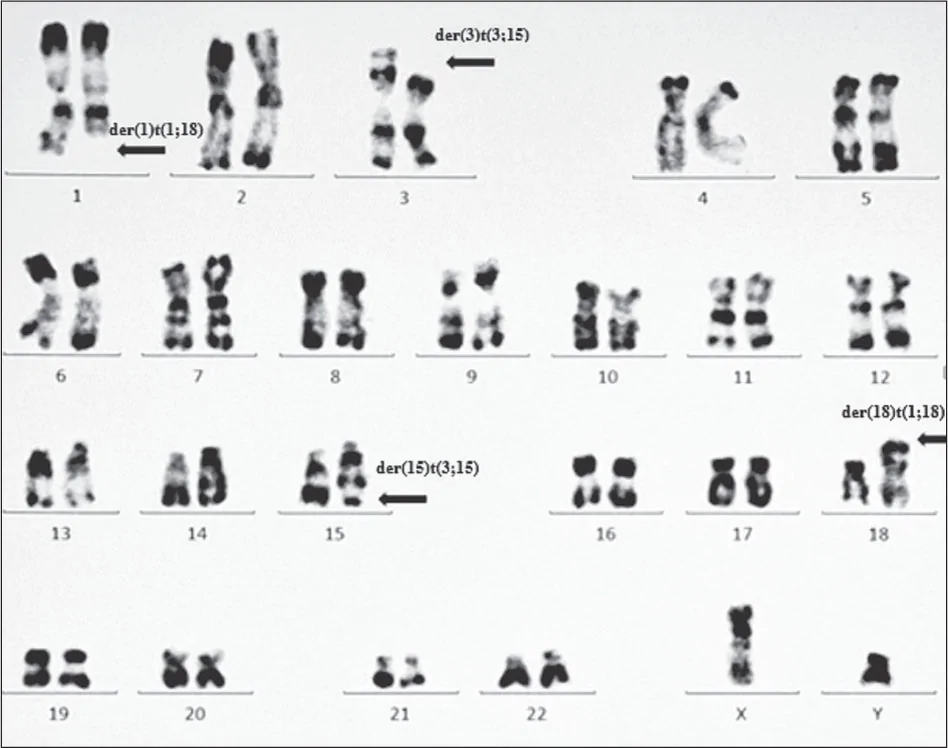
Karyotype image of the husband carried two reciprocal translocations between chromosomes 1 and 18, 3 and 15 (46,XY,t(1;18)(q31;p11),t(3;15)(p22;q25)). Arrows indicate the derivative chromosomes.

The wife exhibited a normal female karyotype: 46,XX (Figure 3).

**Figure 3.**
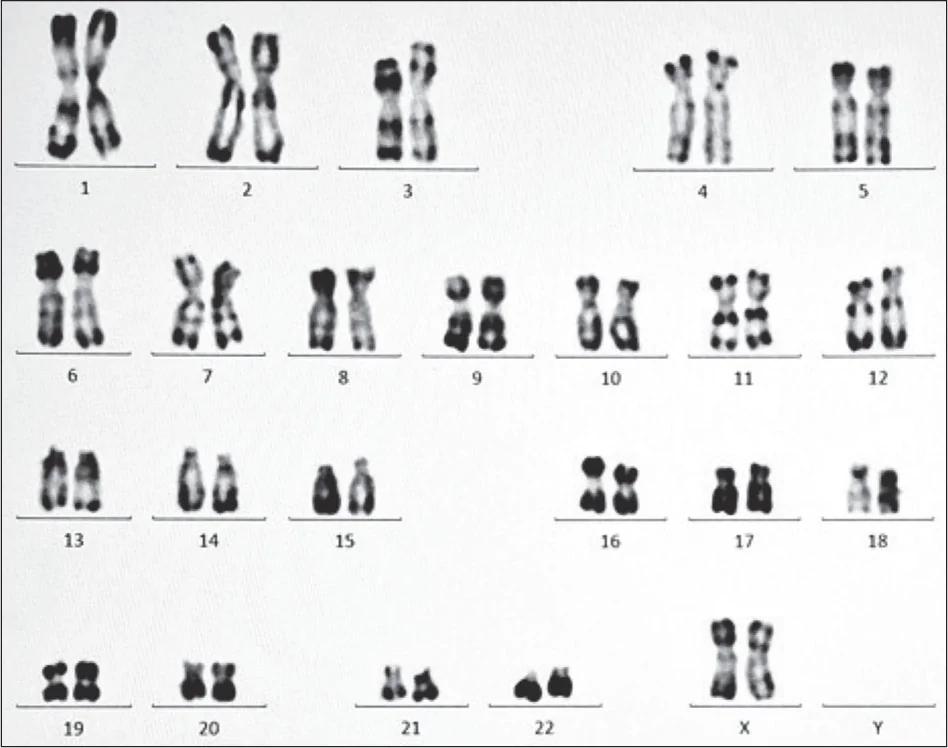
Normal karyotype image of the wife (46,XX)

Karyotyping was not performed on the proband’s daughter, who remains clinically and phenotypically normal, and thus no formal indication for cytogenetic investigation was established.

## DISCUSSION

Double balanced reciprocal translocations belong to the category of complex chromosomal rearrangement (CCR); According to the classification of Kauch et al., these anomalies are characterized as double two-way rearrangements, involving two independent reciprocal translocations [13]. CCR involving two or more translocations are estimated to occur in only 0,0118% of patients with fertility problems, double translocations account for approximately 25% of these rare CCRs [14].

Double two-way CCRs are an important cause of couple infertility, particularly in men with impaired spermatogenesis and those with recurrent pregnancy loss [15]. According to Gorski et al., carriers of balanced CCRs have a 48.3% risk of spontaneous abortion and a 18.4% risk of offspring with malformation syndrome [16]. Furthermore, Madan et al., reviewed 60 cases of balanced CCRs and reported a risk of 50% of miscarriages and 20% of unbalanced karyotypes in liveborn children [17].

In this study, we report an exceptional case of double-balanced reciprocal translocations involving four chromosomes: 1, 3, 15 and 18 (46,XY,t(1;18)(q31;p11),t(3;15) (p22;q25), in a phenotypically normal man explored for recurrent pregnancy loss.

To date, only a few cases of double reciprocal translocations have been reported in the literature (Table 1), making this case a valuable contribution to the understanding of such rearrangement and their reproductive consequences.

**Table 1.** Comparison of double reciprocal translocations in the literature

**Study**	**Chromosomes involved**	**Inheritance pattern**	**Reproductive history**
**Usyal et al.**(18)	3;18 + 13;14	Familial	Male infertility and miscarriages in female carriers
**Pierron et al.**(26)	13;14 + 21;22	De novo	Male infertility
**Barros et al.**(27)	1;19 + 6;14	Familial	4 miscarriages
**Bijlsma et al.**(28)	2;6 + 7;12	Familial	1 miscarriage + child with trisomy 12p
**Vieira et al.**(29)	13;15 + 13;19	De novo	Pregnancy complications + child with dup 15q syndrome and another with Angelman syndrome
**Tabor et al.**(11)	5;7 + 5;12	Familial	3 miscarriages + daughter carrier one translocation
**Ferfouri et al.**(30)	1;16 + 8;9	t(1;16) de novo t(8;9) familial	Normal fertility with 85% unbalanced sperm
**Gupta et al.**(5)	1;3 + 2;14 + 9;11	De novo	Multiple in vitro fertilization failures
**Sinkar and Devi.**(19)	2;12 + 4;16	Familial	2 miscarriages + child carrier one translocation
**Simioni et al.**(10)	3;7 + 15;18	De novo	Proband with developmental delay, hypotonia and dysmorphic features
**Our study**	1;18 + 3;15	Unknown	5 miscarriages + phenotypically normal child

Abbreviation: dup: duplication

The mechanisms leading to the formation of double reciprocal translocations remain poorly understood. Several hypotheses have been proposed, including the simultaneous occurrence of two independent translocations in the same individual. Inheritance from a parent carrying one or both translocations or chromothripsis; a single catastrophic event resulting in multiple chromosomal breaks and rearrangements [18,19]. In this case, the inability to perform cytogenetic analysis on family members precludes determining whether the translocation was inherited or occurred de novo.

The patient exhibited a normal phenotype despite carrying two balanced reciprocal translocations involving fours chromosomes (1, 3, 15, and 18). This is consistent with the literature, which states that carriers of balanced chromosomal rearrangements, such as reciprocal or Robertsonian translocations, are phenotypically normal due to the preservation of their full genetic information, with no visible loss or gain [18,20]. Nevertheless, they have a significantly increased risk of producing unbalanced gametes during meiosis, which can lead to adverse reproductive outcomes such as miscarriages or offspring with congenital anomalies. The involvement of four independent chromosomes in double translocations generates a considerably higher number of possible products of segregation compared to single translocation [7,21]. In carriers of balanced reciprocal translocation, three theoretical segregation patterns might occur during meiosis I: 2:2, 3:1 or 4:0. The two pairs of homologous chromosomes involved form a quadrivalent at the pachytene stage, which is associated with an elevated risk (estimated at 60–70%) of chromosomally unbalanced gametes [22]. Only the alternate segregation mode gives rise to normal or balanced gametes [23].

In this case, where the father carried a double reciprocal independent translocation, we schematized the different outcomes of the various segregation pattern. Analysis of the 2:2 segregation pattern revealed that alternate segregation produces one normal gamete and three balanced gametes. However, adjacent 1 and 2 segregations lead to highly unbalanced gametes. In addition, 3:1 segregation generates gametes with three chromosomes, leading to partial trisomy’s or monosomies, which are usually non-viable. Finally, 4:0 segregation gives rise to nullisomic or disomic gametes for an entire chromosome pair; these are typically lethal during the early embryogenesis (Figure 4). This increases the risk of unbalanced offspring and provides an explanation for the RPL in this couple. The pachytene diagram illustrates the quadrivalent formation involving the chromosomes implicated in the two translocations (Figure 5).

**Figure 4.**
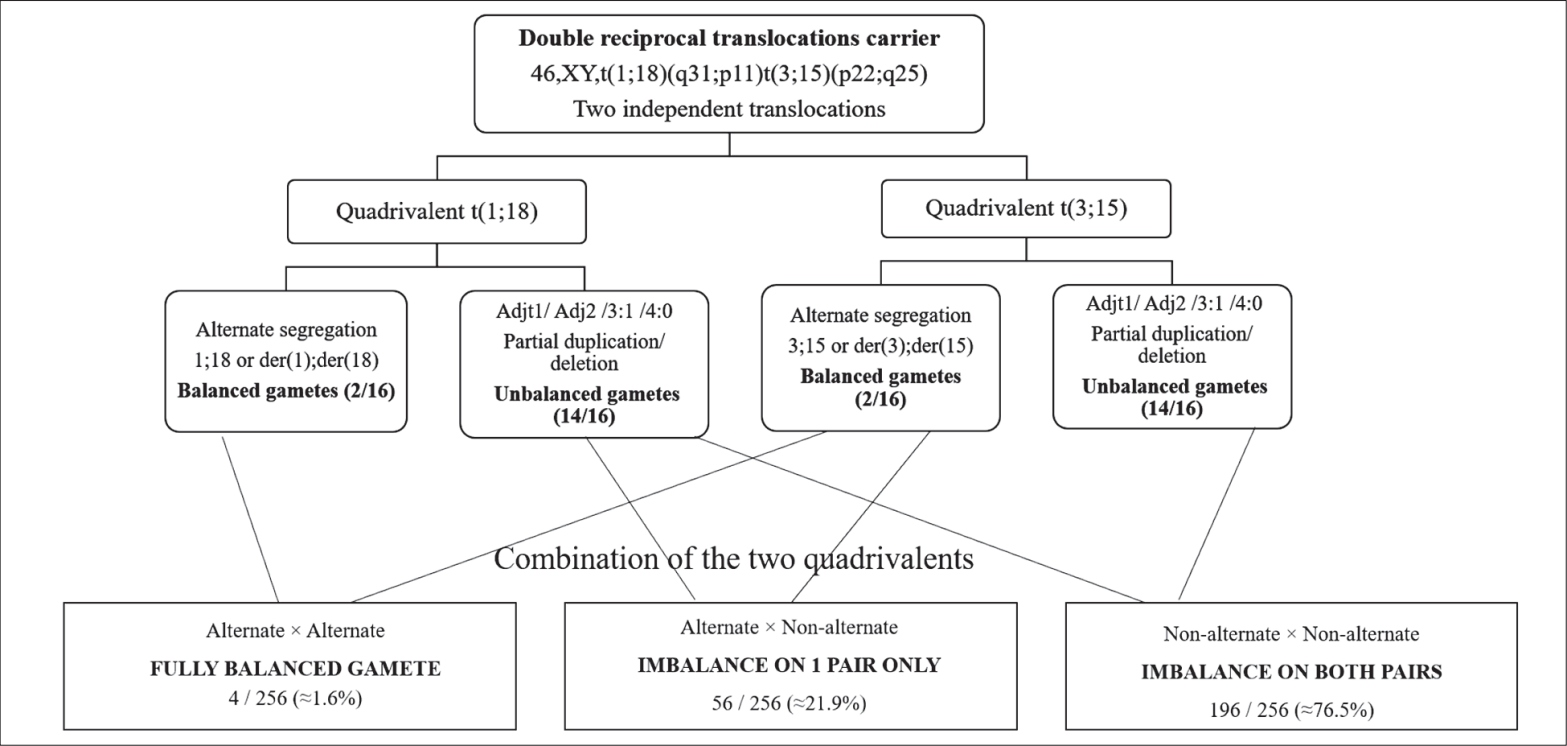
Theoretical gametic outcomes of meiotic segregation in a carrier of two independent balanced reciprocal translocations t(1;18) and t(3;15). Each quadrivalent segregates via 5 modes - alternate, adjacent-1, adjacent-2, 3:1, and 4:0 - producing16 possible gametes per translocation and 256 combined. Only alternate segregation of both quadrivalents produces a balanced gamete (4/256, ≈1.6%); all other combinations result in unbalanced gametes. Segregation-mode frequencies are theoretical and assume independence between the two translocations; in practice, alternate segregation predominates.

**Figure 5.**
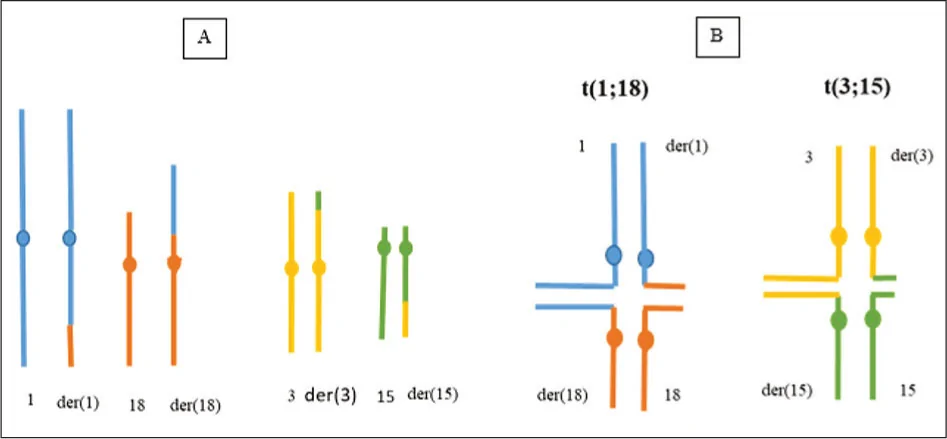
Schematic representation of the double reciprocal translocation in the father. A: Partial karyogram showing the two translocations t(1;18) (q31;p11) and t(3;15)(p22;q25) B: Corresponding diagram of chromosome pairing at the pachytene stage, illustrating the quadrivalent formation.

Interestingly, the couple are the parent of a healthy 9-year-old daughter. One of two possibilities can explain this: either the daughter inherited a normal chromosome from both parents, or she inherited one or both balanced translocations but remained phenotypically normal. Similar observations have been reported in the literature. Uysal et al., reported a remarkable family where a mother carrying a double translocation had three healthy children and only two miscarriages, while her two siblings with the same double translocation were infertile(18). Another study reported a normal child born to a mother carrying a double reciprocal translocation [24]. This illustrates that despite the high reproductive risk, the carriers can give birth to a balanced translocation carrier with a normal phenotype (similar to the parents) or birth to a chromosomally normal, completely healthy infant.

Karyotyping is an inexpensive technique that should be employed as the primary investigative tool for patients with a history of offspring affected by intellectual deficiency or developmental delay, as well as for couples with infertility or adverse obstetric outcomes such as spontaneous or recurrent pregnancy loss[2]. Cytogenetic analysis provides an explanation for the miscarriages and information about the risk for a live-born child with potentially serious anomalies, as well as the risk for future pregnancy, information for members of the extended family who may be at risk and may wish to undergo karyotyping, and availability of prenatal diagnosis in a future pregnancy[25].

However, the possibility of cryptic imbalances undetectable by conventional karyotyping cannot be completely excluded. Advanced molecular techniques such as chromosomal microarray analysis and clinical exome sequencing are recommended to precise mapping of translocation breakpoints and identification of genes located within the unbalanced chromosomal segments [21].

Genetic counseling is crucial in couples with CCRs because of the elevated risk of unbalanced karyotype in offspring. The chance of a healthy live birth depends on the chromosomes and rearrangement type [7]. Reproductive options include prenatal diagnosis (amniocentesis or chorionic villus sampling) with karyotyping, which can determine the fetal chromosomal status in future pregnancies. Preimplantation genetic testing for chromosomal structural rearrangements (PGT-SR) can select and transfer the unaffected embryos [25].

Several limitations of this study must be acknowledged. First, the inability to perform cytogenetic analysis in proband’s parents precludes determining the inheritance pattern of translocation. Second, advanced molecular techniques such as chromosomal microarray analysis (CMA) and fluorescence in situ hybridization (FISH) are recommended to precise characterization of breakpoint. Finally, the proband’s daughter, who is minor and clinically normal was not karyotyped, precluding confirmation of her carrier status despite her asymptomatic phenotype.

## CONCLUSION

This rare case demonstrates the complexity of managing recurrent pregnancy loss and emphasize that parental karyotyping remains indispensable, even in the presence of a phenotypically normal child. Among the possible meiotic segregation patterns, alternate 2:2 segregation can lead either to either chromosomally normal infant or to a balanced translocation carrier with a normal phenotype, explaining the birth of a healthy child despite the high reproductive risk. The exceptional finding of a double reciprocal translocation in a phenotypically normal man with recurrent pregnancy loss contributes to the existing literature on human fertility mechanisms and genetic counseling approaches.

## Abbreviations:

RPL: Recurrent Pregnancy Loss
CCR: Complex Chromosomal Rearrangement
der: derivative chromosome
del: deletion
dup: duplication
CMA: Chromosomal Microarray Analysis
FISH: Fluorescence In Situ Hybridization
